# Breast Ultrasound AI Under Dataset Shift: A Patient-Leakage-Aware Benchmark

**DOI:** 10.3390/diagnostics16101537

**Published:** 2026-05-19

**Authors:** Lulu Wang

**Affiliations:** Department of Engineering, Reykjavik University, 102 Reykjavik, Iceland; lwang381@hotmail.com or luluw@ru.is

**Keywords:** breast ultrasound, artificial intelligence, dataset shift, calibration, diagnostic imaging

## Abstract

**Background:** Artificial intelligence (AI) has shown promise in breast ultrasound image analysis, but most evidence still comes from single-dataset studies. Clinical translation requires evaluation under heterogeneous acquisition and curation conditions. This study presents a patient-leakage-aware, reproducible benchmark for breast ultrasound AI under dataset shift, with emphasis on external generalization, calibration, and confidence-related behavior. **Methods:** A reproducible benchmark framework was developed using patient-level splitting, internal testing, pairwise cross-dataset evaluation, whole-image and region-of-interest (ROI) input strategies, calibration analysis, targeted ROI-margin sensitivity analysis, representative explainable AI visualization, and an auxiliary lesion-versus-normal confidence-based analysis. Four public breast ultrasound datasets (BUSI, BUS-UCLM, BUS-BRA, and BrEaST) were harmonized for a primary benign-versus-malignant lesion classification task. Normal images were excluded from the primary endpoint and used only in auxiliary analyses when sufficient numbers were available. **Results:** Cross-dataset testing was weaker on average than internal testing, with mean raw AUROC decreasing from 0.801 to 0.719 and mean balanced accuracy from 0.723 to 0.635. ROI input improved external performance, especially for the vision transformer, increasing mean external AUROC from 0.666 to 0.805 and mean external balanced accuracy from 0.594 to 0.713 relative to whole-image input. Temperature scaling improved calibration-related metrics, reducing mean external expected calibration error from 0.180 to 0.150 and mean external negative log-likelihood from 0.848 to 0.682. **Conclusions:** This study establishes a reproducible benchmark for evaluating breast ultrasound AI under dataset shift, with explicit attention to patient-level leakage control, external validity, and reliability of predicted probabilities.

## 1. Introduction

Breast ultrasound plays an important role in the evaluation of breast lesions because it is widely available, does not involve ionizing radiation, and is particularly useful in women with dense breast tissue [1,2]. In clinical practice, ultrasound contributes to lesion characterization, biopsy triage, and short-interval follow-up, often complementing mammography and magnetic resonance imaging. Nevertheless, breast ultrasound remains a challenging modality, as image appearance is influenced by operator technique, scanner characteristics, speckle noise, acquisition settings, and lesion context.

Over the past decade, the rapid development of artificial intelligence (AI) techniques has created new opportunities in breast ultrasound diagnostics and analysis [3,4]. In particular, convolutional neural networks (CNNs), transfer learning strategies, and transformer-based architectures such as vision transformers (ViTs) have shown promising performance in lesion detection, segmentation, classification, and decision support [3,4]. Previous breast ultrasound AI studies have also explored segmentation-guided pipelines, peri-lesional or multi-region feature modeling, and transformer-based local-global representation learning to improve benign-versus-malignant discrimination and related diagnostic tasks [5,6]. These advances have strengthened expectations that AI may improve diagnostic consistency and support clinical workflow. However, much of the reported progress has been assessed primarily on the basis of performance within individual datasets rather than evidence of robustness under clinically realistic variation. In addition, direct numerical comparison across published studies remains difficult because datasets, preprocessing methods, train–test splitting strategies, leakage-control procedures, model architectures, thresholding criteria, and validation designs differ substantially.

Clinical translation of breast ultrasound AI depends on more than strong internal test performance. Previous studies have shown that major barriers include incomplete external validation, limited reproducibility, and insufficient evaluation of reliability under real-world conditions [7,8]. The key question, therefore, is not only whether an algorithm can classify images accurately under familiar conditions, but also whether it remains dependable when applied across institutions, devices, and curation pipelines. Patient-leakage-aware evaluation is particularly important in breast ultrasound AI because public datasets may contain multiple images per patient, multiple lesions from the same patient, or heterogeneous patient-identifier structures. If related images are distributed across training and testing partitions, apparent performance may be inflated. Therefore, controlling patient-level partitioning is a necessary step for reliable benchmark interpretation.

These challenges are especially relevant in breast ultrasound, where publicly available datasets differ substantially in scanner composition, image appearance, case mix, annotation format, lesion delineation strategy, and metadata completeness. Resources such as BUS-BRA [9], BrEaST [10], BUS-UCLM [11], and BUSI [12] have markedly improved data availability for reproducible research and expanded the diversity of imaging conditions represented in the literature. Such heterogeneity indicates that apparently similar benign-versus-malignant classification tasks may arise from materially different data-generating environments. Consequently, models trained on one dataset may depend on source-specific cues or curation-related patterns that do not transfer reliably to another dataset. Public datasets are therefore valuable not only for model development, but also for probing the robustness limits of contemporary breast ultrasound AI systems. Because scanner and acquisition-protocol metadata are not consistently available across all public datasets, dataset shift in this study is interpreted as a composite effect of scanner/protocol variation, image appearance, annotation format, cohort structure, case composition, and patient-identifier structure.

Evaluation of breast ultrasound AI remains incomplete when it is based primarily on discrimination metrics such as accuracy, sensitivity, specificity, and the area under the receiver operating characteristic curve (AUROC). Although these measures are fundamental, they do not fully indicate whether a model can be considered trustworthy under dataset shift [13,14,15,16]. A model may preserve moderate discrimination while becoming poorly calibrated or overconfident when applied to unfamiliar data. Such behavior is especially concerning in safety-sensitive diagnostic settings, where confidence estimates may influence threshold selection, downstream decision support, and clinician trust. For this reason, a clinically meaningful evaluation framework should assess not only discrimination, but also confidence reliability and model behavior under uncertainty.

More recently, several research groups have incorporated larger external evaluations, multiple public datasets, peri-lesional context, interpretability analysis, and broader workflow-oriented AI designs [5,6]. These developments reflect increasing recognition of the challenges associated with clinical translation. Nevertheless, most prior studies have remained centered on architectural performance, transfer learning, segmentation quality, or interpretability rather than on an integrated evaluation of robustness under dataset shift. Consequently, relatively few studies have jointly evaluated internal and cross-dataset performance within a leakage-aware framework while also addressing calibration and confidence-related behavior. Many prior reports have demonstrated high internal performance on individual datasets, whereas the present study emphasizes that such performance may not translate directly to cross-dataset settings when patient-level partitioning, calibration, and source–target transfer are evaluated jointly. This study develops a reproducible, patient-leakage-aware benchmark framework for breast ultrasound lesion classification using representative convolutional neural network (CNN) and vision transformer (ViT) baselines across four public datasets. Using this framework, the study investigates three hypotheses: first, that performance under cross-dataset evaluation is lower on average than under internal testing; second, that region-of-interest (ROI) input improves robustness relative to whole-image input under dataset shift; and third, that post hoc temperature scaling improves calibration-related metrics without eliminating the broader loss of external generalization.

The principal contribution of the study is not the introduction of a new network architecture, but the establishment of a structured benchmark for assessing robustness-related behavior under heterogeneous public-data conditions. Specifically, the study provides a patient-leakage-aware benchmark across four public breast ultrasound datasets, evaluates both internal and pairwise external dataset-shift scenarios, compares whole-image and ROI-based input representations for CNN and ViT baselines, reports calibration and confidence-related analyses in addition to discrimination metrics, and includes targeted ROI-margin sensitivity analysis and representative explainable AI visualizations. Accordingly, the study should be interpreted as a benchmark of external generalization, calibration, and input-design effects rather than as evidence that current models are already clinically robust across institutions.

## 2. Materials and Methods

### 2.1. Study Design

This study developed a reproducible, patient-leakage-aware benchmark for evaluating breast ultrasound artificial intelligence under dataset shift. The primary task was lesion classification in a common benign-versus-malignant label space. Images labeled as normal were excluded from the primary endpoint because their availability differed across datasets; when available in sufficient numbers, they were retained for an auxiliary lesion-versus-normal confidence-based analysis. The benchmark was designed to assess model behavior under two settings: internal testing within a dataset and pairwise cross-dataset evaluation, in which models were trained on one dataset and tested on another. This design enabled joint assessment of discrimination, calibration, and confidence-related behavior under heterogeneous public-data conditions.

The benchmark was instantiated using four publicly available breast ultrasound datasets and two representative deep learning baselines, namely a CNN and a ViT. Experiments were repeated using five random seeds and two input strategies, whole-image and region-of-interest (ROI) input, in order to examine the influence of input representation on external generalization. Because ROI-based inference depended on lesion coordinates derived from source annotations, the benchmark is most directly relevant to lesion-level diagnostic decision support after lesion localization rather than to fully autonomous screening workflows. The study was, therefore, designed as a benchmark and robustness investigation with translational relevance, with emphasis on external validity and reliability of predicted probabilities rather than on the introduction of a new network architecture.

**Figure 1:**
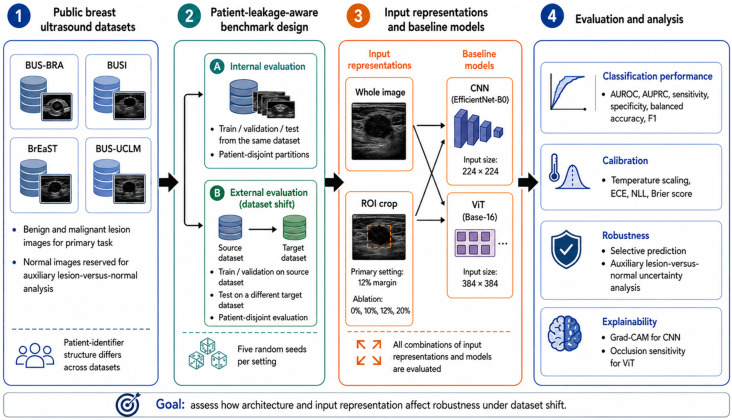
Overview of the patient-leakage-aware benchmarking workflow for breast ultrasound classification across multiple datasets, input representations, models, and evaluation stages. Numbers 1–4 denote the main workflow stages: public breast ultrasound datasets, patient-leakage-aware benchmark design, input representations and baseline models, and evaluation and analysis. Letters A and B indicate the two evaluation settings: internal evaluation and external evaluation under dataset shift, respectively. AUROC, area under the receiver operating characteristic curve; AUPRC, area under the precision–recall curve; ECE, expected calibration error; NLL, negative log-likelihood; CNN, convolutional neural network; ViT, vision transformer; ROI, region of interest.

Figure 1 provides an overview of the study workflow used for the patient-leakage-aware benchmark of breast ultrasound classification under dataset shift. The workflow begins with four public datasets and proceeds through dataset curation, patient-identifier verification, and task definition for benign-versus-malignant lesion classification, with normal cases reserved for auxiliary analyses where applicable. The benchmark then applies patient-level partitioning and constructs both internal and external evaluation scenarios to assess model generalization across datasets. Two input representations, whole-image and ROI-based input, are considered, and two model families, CNN and ViT, are evaluated under the same benchmarking framework. Performance is subsequently assessed using discrimination, calibration, and robustness-related metrics, with additional sensitivity and explainability analyses, including ROI-margin ablation and explainable artificial intelligence visualization.

### 2.2. Benchmark Framework Development

The benchmark framework began with harmonization of source metadata and diagnostic labels across datasets. A harmonized manifest was created to represent dataset membership, patient identity, image location, diagnostic label, and lesion-region information in a common format. Required fields included image path, dataset identifier, patient identifier, diagnostic label, and lesion bounding-box information. Only images labeled as benign, malignant, or normal were accepted, and images with unsupported labels were excluded. For the primary benchmark, only lesion-containing images labeled as benign or malignant were included. When source datasets provided more detailed diagnostic categories, these were collapsed into a common binary label space according to the corresponding dataset documentation. Normal images were excluded from model training and from the primary lesion-classification endpoint because their availability was inconsistent across datasets and would otherwise reduce cross-dataset comparability.

The central safeguard of the benchmark was patient-level leakage control. Images from the same patient were not allowed to appear in more than one partition within a given experimental scenario. Missing patient identifiers were not permitted in publication mode, and benchmarks containing missing or empty patient identifiers were terminated before training. Manifest validation recorded the number of rows and unique patient identifiers per dataset and issued a warning when a patient identifier appeared with both benign and malignant labels within the same dataset. In these cases, all images associated with that identifier were treated as a single indivisible patient-level unit during partitioning. This pattern was treated as a characteristic of public dataset structure rather than as a presumed annotation error, since the available documentation did not clearly indicate whether it reflected multiple lesions within the same patient, dataset-specific labeling structure, or another source characteristic. Mixed-label patient identifiers were retained in the benchmark audit logs and handled conservatively as indivisible patient-level units during partitioning. For stratified patient-level partitioning, each patient identifier required a single stratum assignment. Patient identifiers associated with at least one malignant lesion were assigned to the malignant stratum for partitioning; otherwise, they were assigned to the benign stratum. This rule was used only to allocate patient identifiers to training, validation, or test partitions and did not alter the original image-level diagnostic labels used for model training or evaluation.

No dedicated duplicate or near-duplicate image screening procedure was performed beyond manifest validation and strict patient-level leakage control. These safeguards reduced the risk of patient-level information leakage, but they do not exclude the possibility that visually repeated or highly similar images remained within or across source datasets. In addition, the public datasets did not provide a shared cross-dataset patient identifier. Therefore, although each dataset was handled using its available patient identifiers, the possibility that the same subject appeared in more than one public dataset under different identifiers could not be definitively excluded. Accordingly, the benchmark should be interpreted as patient-leakage-aware rather than as eliminating all forms of image-level redundancy or potential cross-dataset subject overlap. This limitation is important when interpreting the magnitude of both internal and external performance estimates.

The benchmark comprised two principal evaluation settings. For internal testing, each dataset was partitioned at the patient level into training, validation, and test subsets using target fractions of 70%, 10%, and 20%, respectively. Partitioning was performed separately for benign and malignant patient groups and then combined, with both classes represented in all partitions whenever feasible. To ensure that the reported internal results were based on reliable patient-level partitions, a dataset was included in internal evaluation only if it contained at least three patients per class.

For pairwise cross-dataset evaluation, one dataset served as the training source and a different dataset served as the external test target. In these scenarios, the source dataset was partitioned at the patient level into training and validation subsets using target fractions of 85% and 15%, respectively, while the full lesion-containing portion of the target dataset was reserved for external testing. To avoid unstable source training partitions, a source dataset was included in external evaluation only if it contained at least two patients per class. Target datasets were retained only when the lesion-containing test set included both benign and malignant cases.

All experiments were repeated using five random seeds. The same seed list was used for both CNN and ViT models under matched-dataset scenarios and input strategies, enabling a paired comparison across model families. No row-level fallback splitting was used in the reported benchmark. Scenarios that could not satisfy the class-balanced patient-level splitting criteria were skipped and recorded, and scenario-specific split files were exported automatically to support reproducibility. These safeguards were applied to reduce patient-level information leakage, avoid degenerate class partitions, and ensure that reported results were based on interpretable patient-level train, validation, and test splits.

The technical implementation of the benchmark combined fixed reference baselines with two complementary input strategies. Two representative deep learning baselines were evaluated: EfficientNet-B0 as the convolutional baseline and a base-16 vision transformer (ViT-B/16) pretrained on ImageNet at 384-pixel resolution as the transformer baseline. Two input strategies were examined: whole-image input and lesion-centered ROI input. Under both strategies, images were normalized to the range [0, 1], converted to three channels when required, and resized to the dimensions required by the corresponding network. In the ROI setting, crops were generated from lesion bounding-box information with a 12% expansion of the larger lesion dimension to preserve limited peri-lesional context. The ROI condition should therefore be interpreted as an annotation-dependent representation setting that suppresses some nuisance variation by design, rather than as a directly deployment-equivalent alternative to whole-image inference. If ROI information was invalid or cropping failed, the original full image was retained and resized according to a predefined fallback rule. Scenario-specific ROI fallback counts were retained in the benchmark audit logs. Model settings and training hyperparameters are summarized in Table 1.

### 2.3. Datasets

The benchmark was evaluated using four publicly available breast ultrasound datasets: BUS-BRA [9], BrEaST [10], BUS-UCLM [11], and BUSI [12]. The datasets were selected to provide a reproducible public-data benchmark rather than to represent an exhaustive systematic review of all available breast ultrasound datasets. As summarized in Table 2, selection was based on public availability, relevance to breast ultrasound lesion analysis, availability of benign/malignant diagnostic labels, availability of lesion or region-of-interest (ROI) annotation information, and complementary variation in dataset size, patient-identifier structure, normal-image availability, and curation characteristics.

**Figure 2:**
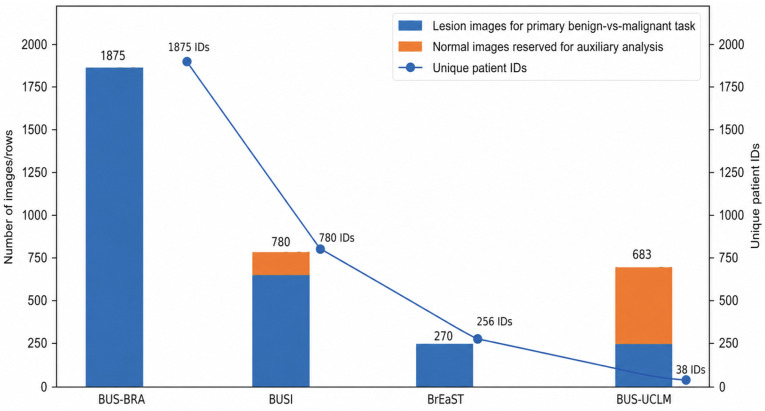
Dataset composition and patient-identifier structure of the included public breast ultrasound datasets. Blue bars show lesion-containing images used for the primary benign-versus-malignant classification task. Orange bars show normal images reserved only for auxiliary confidence-based lesion-versus-normal analysis and not used for primary model training or testing. The line plot shows the number of unique patient identifiers available in each dataset.

Figure 2 summarizes the composition and patient-identifier structure of the four included public breast ultrasound datasets. The figure highlights substantial heterogeneity across datasets in lesion-image counts, availability of normal images, and patient-identifier granularity, which motivated the patient-leakage-aware benchmark design. BUS-BRA contributed 1875 lesion images with 1875 unique patient identifiers, BUSI contributed 647 lesion images and 133 normal images with 780 unique patient identifiers, BrEaST contributed 266 lesion images and 4 normal images with 256 unique patient identifiers, and BUS-UCLM contributed 264 lesion images and 419 normal images but only 38 unique patient identifiers. Thus, the available patient identifiers ranged from near-image-level granularity in some datasets to a markedly more aggregated structure in BUS-UCLM.

BUS-UCLM was also the only dataset in the benchmark with several patient identifiers appearing with both benign and malignant labels, and results involving this dataset were therefore interpreted with particular caution. BUSI and BUS-UCLM were the only datasets with sufficient normal cases for the auxiliary lesion-versus-normal analysis. Additional dataset-structure details, paired ROI-versus-whole-image comparisons, and calibration/safeguard summaries are provided in Supplementary Materials, Tables S1–S3.

Scanner and acquisition-protocol metadata were not consistently available across all public datasets; therefore, scanner composition could not be quantitatively harmonized. Dataset shift was therefore interpreted as a composite of scanner/protocol variation, image appearance, annotation format, cohort structure, case composition, and patient-identifier structure.

### 2.4. Model Baselines, Input Resolution, and ROI Construction

EfficientNet-B0 and ViT-B/16 were evaluated using their standard pretrained input resolutions of 224 × 224 and 384 × 384 pixels, respectively. This choice preserves the conventional operating configuration of each backbone, but it also means that architecture and input resolution are not fully separable factors in the present benchmark. In the ROI setting, the higher ViT resolution may preserve more lesion-boundary and peri-lesional detail after cropping, whereas the lower CNN resolution may impose greater spatial compression. Accordingly, CNN–ViT comparisons should be interpreted as comparisons of representative pretrained baseline configurations rather than as a resolution-controlled architecture-only comparison.

Two input strategies were examined: whole-image input and lesion-centered ROI input. Under both strategies, images were normalized to the range [0, 1], converted to three channels when required, and resized to the dimensions required by the corresponding network. No additional ImageNet mean and standard deviation normalization was applied after scaling to [0, 1]; this preprocessing choice was kept fixed across both model families to maintain a consistent grayscale ultrasound image-processing pipeline. In the ROI setting, crops were generated from lesion bounding-box information with a 12% expansion of the larger lesion dimension. This margin was prespecified to retain immediate peri-lesional tissue and lesion-boundary context while limiting the inclusion of unrelated anatomy, scanner overlays, and source-specific image framing. A 0% margin may crop diagnostically relevant boundary context, whereas substantially larger margins may reintroduce the same background and acquisition cues that ROI input is intended to suppress. The 12% margin should therefore be interpreted as a fixed design choice for a controlled benchmark, not as an optimized hyperparameter selected from external test performance. If ROI information was invalid or cropping failed, the original full image was retained and resized according to a predefined fallback rule, and scenario-specific fallback counts were retained in the benchmark audit logs.

To address the potential influence of the selected ROI expansion margin, an additional targeted sensitivity analysis was performed using ROI margins of 0%, 10%, 12%, and 20%. This analysis was restricted to four representative external dataset-shift scenarios involving all four datasets across source and target roles (BUS-UCLM to BUSI, BUSI to BUS-BRA, BUS-BRA to BUSI, and BrEaST to BUS-UCLM), the three targeted ROI-margin sensitivity seeds, and both model families. The purpose of this analysis was not to replace the complete 320-run benchmark, but to determine whether the original 12% ROI margin materially affected the external-performance interpretation.

### 2.5. Evaluation and Statistical Analysis

The evaluation used external AUROC for benign-versus-malignant classification, computed from the raw model outputs, as the primary performance measure. Secondary discrimination metrics included area under the precision–recall curve (AUPRC), sensitivity, specificity, balanced accuracy, and F1 score, together with the reliability-oriented metrics expected calibration error (ECE) and negative log-likelihood (NLL). For threshold-dependent metrics, predictions were classified as malignant when the predicted malignant-class posterior probability was ≥0.5. This threshold was fixed a priori and was not tuned on internal or external test data. ECE was computed as the sample-size-weighted average of the absolute difference between mean predicted confidence and empirical accuracy across 10 equal-width bins spanning [0, 1]. All metrics were computed for both raw and temperature-scaled outputs. Raw results were treated as the primary findings under dataset shift, whereas calibrated results were reported as supplementary reliability analyses. Detailed scenario-level paired comparisons of ROI-based and whole-image input under cross-dataset evaluation are provided in Supplementary Materials Table S2.

Calibration analyses were performed alongside the standard discrimination evaluation. Post hoc temperature scaling was fitted using only the source-domain validation partition and then applied unchanged to the corresponding test predictions. The calibration procedure was performed separately for each completed combination of dataset scenario, random seed, model family, and input strategy. Temperature scaling was attempted only when the validation set contained at least 40 examples, including at least 10 malignant and 10 benign cases. If these requirements were not met, calibration was not applied and the temperature parameter was set to 1.0. To avoid degenerate solutions, temperature optimization was constrained to the interval [0.5, 5.0] using one-dimensional bounded minimization of validation negative log-likelihood. Calibration eligibility counts, audited safeguard checks, and context for the auxiliary lesion-versus-normal confidence-based analysis are summarized in Supplementary Materials Table S3.

For the auxiliary lesion-versus-normal analysis, uncertainty was quantified as the complement of the maximum predicted posterior probability. This quantity is a maximum-softmax-based confidence proxy rather than a dedicated uncertainty-estimation method, so the corresponding analysis was treated as an auxiliary confidence-based assessment rather than as a definitive open-set detection benchmark. These results were reported only when the number of normal cases was at least 20; otherwise, the corresponding metrics were suppressed and recorded as unavailable.

In addition to descriptive means, 95% confidence intervals were estimated for the principal aggregate benchmark metrics using train–test scenario-level summaries. The inferential unit for aggregate comparisons was the train–test scenario rather than the individual seed-level run, as repeated random seeds within the same scenario do not represent independent realizations of dataset shift. For each experimental configuration, results were first averaged across the five random seeds within each scenario, and confidence intervals for the across-scenario mean were then computed using the t distribution. For bounded measures such as AUROC and balanced accuracy, the displayed confidence intervals were truncated to the interval [0, 1] for interpretability. Paired comparisons were performed on scenario-level differences using the Wilcoxon signed-rank test. The confidence intervals reported in the main tables therefore summarize across-scenario uncertainty. A sensitivity analysis was also performed by recomputing aggregate summaries after excluding all scenarios involving BUS-UCLM.

### 2.6. Explainable AI Analysis

To complement the quantitative benchmark results, an explainable AI (XAI) analysis was performed for representative external dataset-shift scenarios. For the CNN model, Grad-CAM was used to visualize image regions contributing most strongly to the predicted malignant class. For the ViT model, model-agnostic occlusion sensitivity was used to identify spatial regions that most influenced the model output. XAI examples were generated from selected saved models under the primary experimental setting to provide qualitative insight into how architecture and input representation influenced model behavior under dataset shift.

### 2.7. Implementation Environment

All experiments were implemented in MATLAB R2025a (The MathWorks, Inc., Natick, MA, USA); using Deep Learning Toolbox, Image Processing Toolbox, and Statistics and Machine Learning Toolbox, with GPU acceleration enabled through Parallel Computing Toolbox when available. In the present implementation, experiments were conducted on a workstation equipped with an NVIDIA GeForce RTX 5090 Laptop GPU (NVIDIA Corporation, Santa Clara, CA, USA).

During the preparation of this manuscript, the author used ChatGPT 5.4 to assist with editing and improving the clarity of the text. The author reviewed and edited the output and takes full responsibility for the content of this publication.

## 3. Results

**Figure 3:**
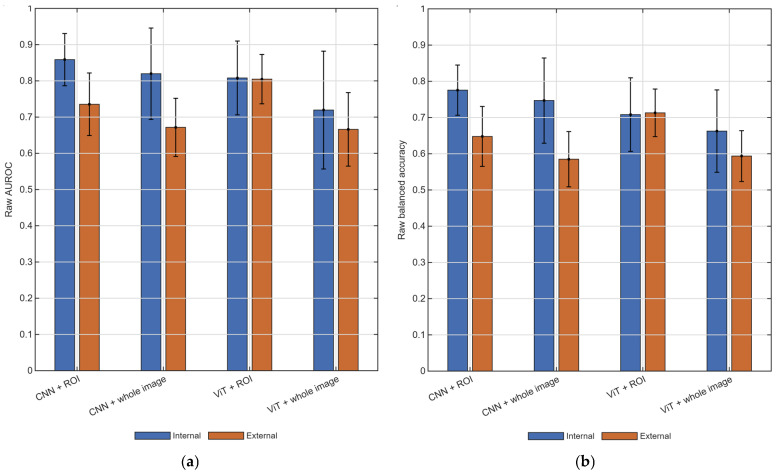
Overall raw classification performance under internal and external evaluation. (**a**) Mean raw AUROC. (**b**) Mean raw balanced accuracy. Bars show scenario-level means averaged over five random seeds; error bars show seed-level standard deviations. AUROC, area under the receiver operating characteristic curve; CNN, convolutional neural network; ViT, vision transformer; ROI, region of interest.

Across four internal and twelve pairwise cross-dataset evaluation scenarios, five random seeds, two input strategies, and two model families, the benchmark yielded 320 completed train–test runs. Unless otherwise stated, the values reported below and in Table 3 are means aggregated from scenario-level summaries over the five random seeds. As shown in Figure 3 and Table 3, internal performance exceeded cross-dataset performance on average; in Figure 3, the error bars represent seed-level standard deviations and therefore summarize within-scenario variation rather than across-scenario uncertainty. Mean raw AUROC decreased from 0.801 internally to 0.719 under cross-dataset evaluation, and mean raw balanced accuracy decreased from 0.723 to 0.635. These findings indicate that single-dataset evaluation can substantially overestimate likely performance under heterogeneous external conditions, although the magnitude of degradation depended strongly on the specific source-target pairing. Confidence intervals based on scenario-level summaries supported the same overall interpretation. Under cross-dataset evaluation, mean AUROC was 0.735 (95% CI 0.681–0.790) for the CNN with ROI input and 0.805 (95% CI 0.762–0.848) for the ViT with ROI input, compared with 0.672 (95% CI 0.621–0.723) and 0.666 (95% CI 0.602–0.731), respectively, for the corresponding whole-image configurations. Similar patterns were observed for balanced accuracy.

BUS-UCLM was treated as a structurally atypical but informative stress-test dataset because of its constrained patient-level structure and the presence of patient identifiers associated with both benign and malignant labels. To assess whether the main findings depended disproportionately on this dataset, aggregate summaries were recomputed after excluding all scenarios involving BUS-UCLM. The principal conclusions remained unchanged: cross-dataset performance remained lower than internal performance on average, and ROI input remained superior to whole-image input for both model families. The magnitude of the aggregate degradation was attenuated, however, with overall mean external area under the receiver operating characteristic curve increasing from 0.719 to 0.761 and overall mean external balanced accuracy increasing from 0.635 to 0.673. These findings indicate that BUS-UCLM contributed materially to benchmark difficulty but did not determine the central conclusions of the study. Additional dataset characteristics are summarized in Supplementary Materials Table S1, detailed paired ROI-versus-whole-image comparisons are reported in Supplementary Materials Table S2, and calibration eligibility and safeguard details are summarized in Supplementary Materials Table S3.

Audit checks of the external ROI scenarios with BrEaST as the source dataset confirmed the absence of patient overlap between source-domain training and validation partitions and target-domain lesion test sets in all evaluated seeds. In scenarios with auxiliary normal-image analysis, the normal-image partitions were also disjoint from the source-domain partitions. In the audited ROI scenario training on BrEaST and testing on BUSI, the BUSI lesion-containing test set contained 647 cases and the auxiliary normal-image set contained 133 cases, with no patient overlap between them. In the corresponding ROI scenario training on BrEaST and testing on BUS-UCLM, the lesion-containing test set contained 264 cases and the auxiliary normal-image set contained 419 cases; however, these two target partitions were not patient-disjoint and were therefore interpreted cautiously.

A consistent effect of input strategy was observed. ROI input outperformed whole-image input for both model families, particularly under cross-dataset evaluation (Figure 3; Table 3). For the CNN, external area under the receiver operating characteristic curve increased from 0.672 to 0.735 and balanced accuracy from 0.585 to 0.648. For the ViT, external area under the receiver operating characteristic curve increased from 0.666 to 0.805 and balanced accuracy from 0.594 to 0.713. Among all evaluated configurations, the ViT with ROI input achieved the strongest overall cross-dataset performance. Scenario-level paired comparisons confirmed this advantage. For the CNN, the mean paired gain under cross-dataset evaluation was 0.064 in area under the receiver operating characteristic curve (95% CI 0.025–0.102; Wilcoxon *p* = 0.009) and 0.063 in balanced accuracy (95% CI 0.039–0.087; *p* < 0.001). For the ViT, the corresponding gains were 0.139 in area under the receiver operating characteristic curve (95% CI 0.101–0.176; *p* < 0.001) and 0.119 in balanced accuracy (95% CI 0.100–0.138; *p* < 0.001). Under ROI input, the ViT also outperformed the CNN on average in cross-dataset area under the receiver operating characteristic curve and balanced accuracy, whereas no clear architecture-level advantage was observed in the whole-image setting.

**Figure 4:**
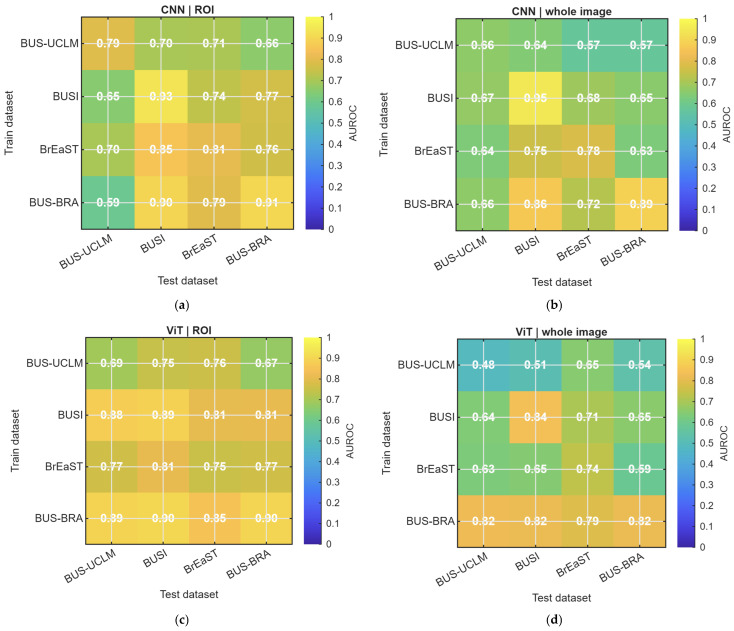
Mean raw AUROC for each train–test dataset pair. Rows are training datasets and columns are test datasets. Diagonal cells show internal evaluation; off-diagonal cells show external evaluation. (**a**) CNN with ROI input. (**b**) CNN with whole-image input. (**c**) ViT with ROI input. (**d**) ViT with whole-image input. CNN, convolutional neural network; ViT, vision transformer; ROI, region of interest.

The pairwise area under the receiver operating characteristic curve heatmaps (Figure 4) showed that cross-dataset transfer was heterogeneous and asymmetric. Several ROI-based transfers achieved strong discrimination, particularly when BUS-BRA served as the training source. As summarized in Table 4, the strongest external setting was obtained by training on BUS-BRA and testing on BUSI with the ViT using ROI input, which achieved an area under the receiver operating characteristic curve of 0.905 and a balanced accuracy of 0.815. By contrast, the weakest transfers were dominated by whole-image input and frequently involved BUS-UCLM as the training source. The lowest external area under the receiver operating characteristic curve was observed when training on BUS-UCLM and testing on BUSI with the ViT using whole-image input, which yielded an area under the receiver operating characteristic curve of 0.513 and a balanced accuracy of 0.513. These results indicate that the effect of dataset shift depended strongly on the specific source–target pairing.

**Figure 5:**
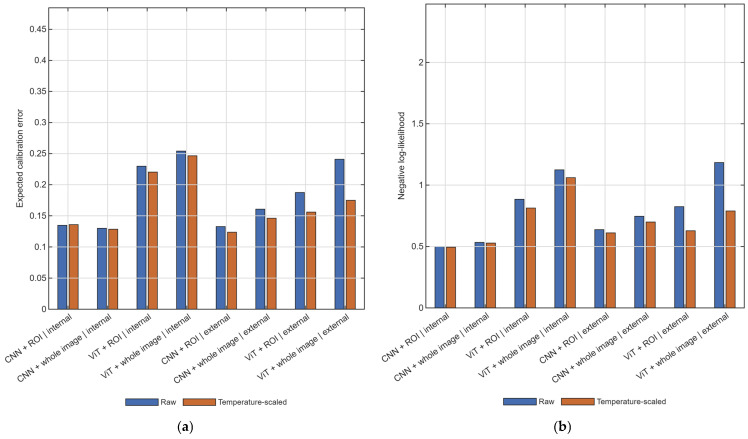
Calibration summary across internal and external evaluation settings. (**a**) Expected calibration error before and after temperature scaling. (**b**) Negative log-likelihood before and after temperature scaling. Temperature scaling was applied only when source-domain validation eligibility criteria were met. CNN, convolutional neural network; ViT, vision transformer; ROI, region of interest.

Figure 5 summarizes calibration across internal and external evaluation settings. Temperature scaling improved confidence-related metrics in both settings, with larger gains under external validation. Mean external expected calibration error decreased from 0.180 to 0.150, and mean external negative log-likelihood decreased from 0.848 to 0.682 after calibration. The largest improvement was observed for the ViT with whole-image input in the external setting, where negative log-likelihood decreased from 1.184 to 0.789. Internal improvements were smaller, with mean expected calibration error decreasing from 0.187 to 0.183 and mean negative log-likelihood decreasing from 0.760 to 0.724. These aggregate improvements should be interpreted together with the calibration-eligibility criteria, rather than as though temperature scaling had been estimated under identical conditions in every run. Across the completed benchmark runs, the predefined validation-set criteria for temperature scaling were satisfied in 44 of 80 internal runs (55.0%) and 180 of 240 external runs (75.0%). Additional eligibility and safeguard details are summarized in Supplementary Materials Table S3.

**Figure 6:**
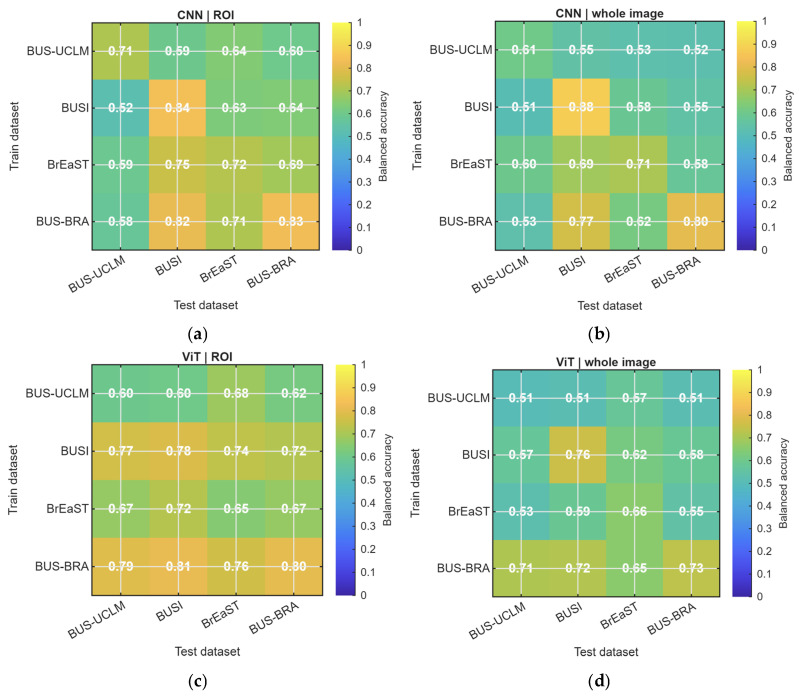
Mean raw balanced accuracy for each train–test dataset pair. Rows are training datasets and columns are test datasets. Diagonal cells show internal evaluation; off-diagonal cells show external evaluation. (**a**) CNN with ROI input. (**b**) CNN with whole-image input. (**c**) ViT with ROI input. (**d**) ViT with whole-image input. CNN, convolutional neural network; ViT, vision transformer; ROI, region of interest.

Figure 6 presents the pairwise balanced-accuracy heatmaps. The balanced-accuracy patterns broadly paralleled the AUROC results, although the absolute values were generally lower, as expected for a threshold-dependent metric. ROI-based models maintained more favorable and more stable balanced accuracy across external targets, whereas whole-image models more often approached the range of 0.5 to 0.6, particularly in transfers involving BUS-UCLM. This effect was most evident for the vision transformer, for which ROI input consistently outperformed whole-image input across most dataset pairs.

**Figure 7:**
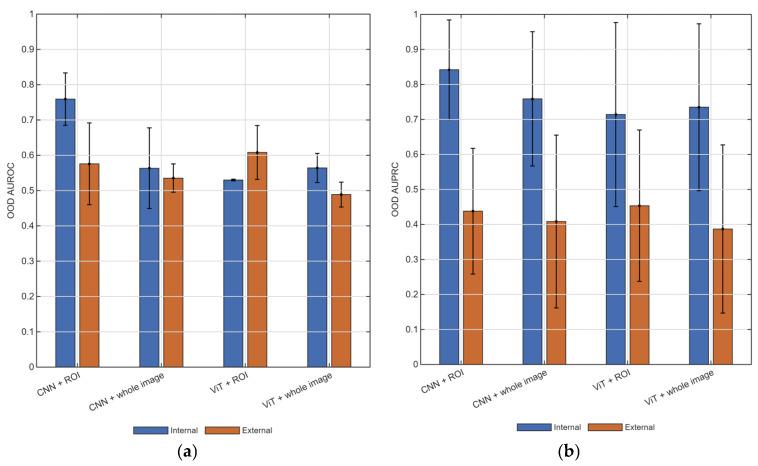
Auxiliary lesion-versus-normal confidence-based analysis. (**a**) AUROC. (**b**) AUPRC. AUPRC, area under the precision–recall curve; AUROC, area under the receiver operating characteristic curve; CNN, convolutional neural network; ViT, vision transformer; ROI, region of interest.

The auxiliary lesion-versus-normal confidence-based analysis (Figure 7) showed modest separation overall and weaker performance under external than internal evaluation. Although some ROI-based configurations showed partial ability to distinguish lesion-containing from normal images, the magnitude and consistency of this effect were limited. Because sufficient normal cases were available only for BUSI and BUS-UCLM, these analyses were narrower in scope than the primary benign-versus-malignant benchmark. Moreover, in the audited BrEaST-to-BUS-UCLM ROI setting, lesion-containing and auxiliary normal target partitions were not patient-disjoint, which further warrants caution in interpretation. Predictive confidence from the present discriminative classifiers therefore provided only modest additional safety-relevant information and should not be interpreted as establishing a reliable open-set rejection.

A sensitivity analysis excluding all BUS-UCLM scenarios is summarized in Table 5. The principal conclusions remained unchanged: external performance remained lower than internal performance on average, and ROI input remained superior to whole-image input for both model families. The magnitude of the aggregate degradation was attenuated after excluding BUS-UCLM, indicating that this dataset contributed materially to benchmark difficulty but did not determine the overall interpretation of the study.

ROI crops were generated from lesion bounding-box information with 12% expansion of the larger lesion dimension to preserve limited peri-lesional context. This margin was selected to retain immediate tissue adjacent to the lesion boundary while limiting inclusion of broader background and dataset-specific image context. To assess whether this choice materially affected the benchmark interpretation, a targeted ROI-margin sensitivity analysis was performed using expansion margins of 0%, 10%, 12%, and 20%.

In the targeted ROI-margin sensitivity analysis, external performance was broadly stable across ROI expansion margins of 0%, 10%, 12%, and 20% (Table 6; Supplementary Materials Tables S4 and S5). For the CNN, mean AUROC values across the representative external scenarios were 0.778, 0.772, 0.751, and 0.738, respectively; the corresponding balanced accuracy values were 0.655, 0.658, 0.656, and 0.643. For the ViT, mean AUROC values were 0.834, 0.826, 0.816, and 0.806, with balanced accuracy values of 0.706, 0.708, 0.698, and 0.704. Paired comparisons against the primary 12% margin did not show statistically significant differences in AUROC or balanced accuracy for either model family. These findings indicate that the ROI-based conclusions were not driven by the exact 12% expansion margin.

**Figure 8:**
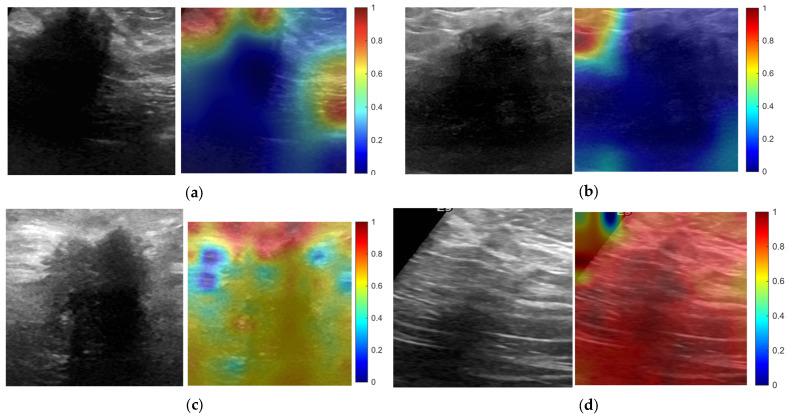
Representative explainable AI visualizations under external dataset shift. Representative examples are shown for selected external transfer scenarios under the primary ROI setting with 12% expansion. (**a**) CNN Grad-CAM visualization for BUS-BRA → BUSI. (**b**) CNN Grad-CAM visualization for BUS-UCLM → BUSI. (**c**) ViT occlusion-sensitivity visualization for BUS-BRA → BUSI. (**d**) ViT occlusion-sensitivity visualization for BUS-UCLM → BUSI. For each panel, the grayscale image is shown next to the corresponding relevance map. CNN models were visualized using Grad-CAM, whereas ViT models were visualized using model-agnostic occlusion sensitivity. Warmer colors indicate higher relative relevance or sensitivity.

Representative explainable AI visualizations are shown in Figure 8 for selected external dataset-shift scenarios under the primary ROI setting with 12% expansion. CNN models were visualized using Grad-CAM, whereas ViT models were visualized using model-agnostic occlusion sensitivity. The examples show that spatial relevance patterns varied across model family and source–target setting, with CNN Grad-CAM and ViT occlusion-sensitivity maps displaying different distributions of image evidence under ROI-based external testing. These qualitative findings support the interpretation that robustness under dataset shift depends on the interaction between architecture and input representation.

## 4. Discussion

This study evaluated breast ultrasound artificial intelligence under dataset shift using a patient-leakage-aware benchmark with internal testing, pairwise cross-dataset evaluation, calibration analysis, ROI-margin sensitivity analysis, representative XAI visualization, and an auxiliary confidence-based lesion-versus-normal assessment across four public datasets. Performance was lower under external validation than under internal testing on average, indicating that single-dataset evaluation can overestimate likely real-world performance. At the same time, performance varied substantially across source–target pairs, showing that robustness was strongly dataset dependent rather than adequately characterized by any single external cohort. The main contribution of the work is therefore methodological and translational: it provides a structured framework for assessing robustness-related behavior under heterogeneous public-data conditions rather than evidence of robustness sufficient for clinical deployment.

Recent breast ultrasound AI studies and reviews have reported encouraging performance for lesion classification, segmentation, diagnosis, and decision support, reflecting rapid progress in convolutional neural networks, transfer learning, transformer-based architectures, and multi-region feature modeling [3,4,5,6]. These studies have helped establish the potential value of AI in breast ultrasound analysis. However, much of the existing literature remains focused on architectural performance, internal validation, or task-specific results within individual datasets. The present benchmark complements this literature by emphasizing external generalization, patient-level leakage control, calibration, and reliability under dataset shift. The findings are also consistent with broader concerns in medical imaging AI that models with high internal validity may still have limited external robustness, reproducibility, and deployment readiness when applied to data from different institutions, scanners, operators, annotation protocols, or curation pipelines [7,8,16].

Direct numerical comparison with prior breast ultrasound AI studies remains difficult because published studies differ in dataset selection, train–test splitting strategy, patient-level leakage control, task definition, model architecture, and external validation design. Many prior reports have demonstrated high internal performance on individual datasets, whereas the present study emphasizes that such performance may not translate directly to cross-dataset settings when patient-level partitioning, calibration, and source–target transfer are evaluated jointly. A contextual comparison with related breast ultrasound AI, public-dataset, uncertainty-quantification, and medical-imaging AI translation studies is provided in Supplementary Materials Table S6.

The clearest technical result was the advantage of ROI input over whole-image input, particularly for the vision transformer. This suggests that lesion-focused input can reduce reliance on contextual features that vary across datasets, such as framing, surrounding anatomy, and machine-specific overlays. This observation is consistent with prior work suggesting that lesion-centered and peri-lesional information can contribute to breast ultrasound classification performance [5,6]. However, this advantage should be interpreted in light of the annotation-dependent design of the ROI condition. Since ROI-based inference depended on lesion coordinates derived from public dataset annotations, the benchmark is most directly relevant to lesion-level diagnostic decision support and robustness assessment after lesion localization, rather than to fully autonomous screening deployment. Accordingly, the observed ROI benefit should not be interpreted as a universal claim that ROI-based pipelines are inherently superior across all clinical workflows.

The targeted ROI-margin sensitivity analysis further supports the robustness of the ROI-based interpretation. The original 12% expansion margin was selected to preserve limited peri-lesional context while avoiding excessive inclusion of broader image background and dataset-specific artifacts. In the targeted analysis, 0%, 10%, 12%, and 20% ROI margins were compared across representative external dataset-shift scenarios involving all four datasets. No tested alternative margin differed significantly from the primary 12% margin for AUROC or balanced accuracy in either model family. These findings indicate that the main ROI-based conclusions were not driven by the exact 12% margin. The 12% setting was therefore retained as the primary ROI configuration because it provides limited peri-lesional context while reducing the amount of surrounding background.

The contrasting behavior of the two baseline architectures is also informative. The vision transformer achieved the strongest overall external performance when combined with ROI input, yet it was among the weakest configurations in the whole-image setting. This indicates that robustness depended less on model family alone than on the interaction between architecture and input representation. Given that only two representative baseline families were examined, these findings should be interpreted as controlled benchmark evidence rather than as a definitive ranking of convolutional and transformer-based approaches. Direct CNN–ViT comparisons should also be interpreted cautiously because the two pretrained baselines used their standard architecture-specific input resolutions; therefore, the benchmark compares representative pretrained configurations rather than isolating architecture from input size.

The explainable AI analysis provided qualitative support for the quantitative findings. Representative CNN Grad-CAM maps and ViT occlusion-sensitivity maps showed different spatial relevance patterns under ROI-based external testing. CNN examples tended to show localized relevance near lesion or peri-lesional regions, whereas ViT occlusion-sensitivity maps showed broader and more spatially distributed sensitivity patterns. These qualitative findings are consistent with the interpretation that robustness under dataset shift depends on the interaction between architecture and input representation. However, these visualizations should be interpreted cautiously, as Grad-CAM and occlusion sensitivity provide supportive qualitative evidence rather than definitive causal explanations of model decision-making.

Calibration analysis added a complementary perspective on reliability. Temperature scaling improved expected calibration error and negative log-likelihood in both internal and external settings, with larger gains under external validation. This finding is consistent with the recent literature emphasizing that discrimination metrics alone are insufficient for trustworthy clinical AI, particularly when models are deployed under distribution shift [14,15]. A model may retain moderate AUROC while producing poorly calibrated or overconfident probabilities, which is problematic for threshold-based decision support, risk communication, and clinician trust. This indicates that some confidence misalignment induced by dataset shift can be corrected after training, but calibration did not alter the broader pattern of reduced external discrimination. Its interpretation also depends on calibration eligibility, because temperature scaling was not estimable in every run and defaulted to 1.0 when predefined validation-set criteria were not satisfied. Thus, calibration improved reliability of predicted probabilities in eligible settings but did not resolve the underlying generalization problem. Calibration should therefore be considered a complementary reliability assessment rather than a substitute for external validation.

The auxiliary lesion-versus-normal confidence-based analysis should likewise be interpreted cautiously. Only BUSI and BUS-UCLM contained sufficient normal cases for this analysis, and the observed separation was modest, particularly under external evaluation. In addition, the Supplementary Materials audit showed that the lesion-containing and auxiliary normal partitions were not patient-disjoint in the BrEaST-to-BUS-UCLM ROI scenario. The corresponding results therefore provide limited, auxiliary safety-oriented information and should not be interpreted as a definitive uncertainty or open-set detection benchmark. These findings suggest that maximum-softmax-based confidence provides limited safety-oriented information in this setting. Future work should evaluate dedicated uncertainty-estimation, out-of-distribution detection, and abstention methods within the same leakage-aware framework.

The BUS-UCLM results warrant particular caution. This dataset had the smallest patient-level cohort and the most unusual patient-label structure, with several identifiers associated with both benign and malignant labels. Supplementary Materials Table S1 further shows that the benchmark datasets differed materially in patient-identifier structure, ranging from 38 unique identifiers in BUS-UCLM to near-row-level identifier granularity in BUSI and BUS-BRA. These structural differences are relevant to effective sample size, partition behavior, and cross-dataset comparability, and they likely contributed to the difficulty of transfers involving BUS-UCLM. The sensitivity analysis excluding BUS-UCLM attenuated the average external-performance decline while preserving the principal conclusions, supporting the overall interpretation of the benchmark while indicating that BUS-UCLM functioned as a disproportionately difficult source-target condition. Differences in source-dataset size and patient-level structure may also have influenced the stability of model learning. The pairwise benchmark intentionally preserved these differences rather than equalizing training-set size, because they reflect the practical difficulty of transferring models trained on one public dataset to another.

Several limitations should be acknowledged. The benchmark relied on public datasets, which supports reproducibility and transparency but limits control over pathology confirmation, acquisition standardization, metadata completeness, scanner settings, operator technique, annotation standards, patient selection, and curation pipelines. Therefore, the benchmark should be interpreted as a reproducible public-data robustness study rather than as definitive evidence of clinical deployment readiness. Inclusion of an institutionally independent in-house dataset would strengthen clinical validation; however, private datasets may reduce full reproducibility unless they can be released or audited under a transparent protocol. Only two representative model families were evaluated, and the ROI pipeline depended on lesion annotations that may not be available in all workflows. Calibration and confidence-based analyses were constrained by the size of the validation and normal-case subsets, especially in smaller datasets. The benchmark also focused on binary benign-versus-malignant classification rather than broader clinical tasks such as lesion detection, BI-RADS support, biopsy recommendation, short-interval follow-up, reader assistance, or integration with multimodal patient information. In addition, although patient-level leakage was controlled explicitly, no separate duplicate or near-duplicate image screening step was performed, so some residual image-level redundancy cannot be fully excluded and may influence the apparent magnitude of performance. For this reason, the present benchmark should be interpreted as patient-leakage-aware rather than as eliminating all possible forms of image-level duplication or curation-related redundancy.

Future work should evaluate multi-source training and domain-generalization approaches within the same framework, examine abstention or open-set strategies for safety-oriented triage, and validate findings on institutionally independent or multi-center cohorts, ideally including standardized acquisition metadata, pathology confirmation, BI-RADS assessment, reader–AI comparison, and prospective or externally audited evaluation. Future benchmark releases should also incorporate explicit duplicate and near-duplicate screening and expanded appendix reporting of per-dataset patient-level structure, class composition, and scenario-level sample sizes. The framework should also be extended to BI-RADS prediction, automated lesion localization, calibrated triage, and reader–AI interaction to better reflect clinical breast ultrasound workflows.

## 5. Conclusions

This study establishes a reproducible, patient-leakage-aware benchmark for evaluating breast ultrasound lesion classification under dataset shift across heterogeneous public datasets and underscores the importance of assessing performance beyond single-dataset evaluation alone. The benchmark highlights the need to consider dataset heterogeneity, patient-level leakage control, input design, and reliability of predicted probabilities when evaluating breast ultrasound AI in settings relevant to clinical translation. Its principal contribution is to provide a structured framework for assessing external generalization, calibration, and confidence-related behavior under heterogeneous public-data conditions. The results show that cross-dataset performance is lower on average than internal performance, that ROI-based input can improve external performance within an annotation-dependent lesion-level setting, and that temperature scaling improves reliability metrics without removing the broader loss of external discrimination. Accordingly, the present findings should be interpreted as benchmark evidence about robustness-related behavior rather than as proof of robustness sufficient for clinical deployment across institutions.

## Figures and Tables

**Table 1:** CNN and ViT benchmark architectures and training settings.

Setting	CNN Baseline	ViT Baseline
Model family	Convolutional neural network	Vision transformer
Backbone	EfficientNet-B0	ViT base-16
Pretraining	ImageNet-pretrained	ImageNet-pretrained
Classification task	Binary benign vs. malignant	Binary benign vs. malignant
Input modes	Whole image; ROI-based crop	Whole image; ROI-based crop
Input image size	224 × 224 × 3	384 × 384 × 3
Optimizer	Adam	Adam
Initial learning rate	$1\times 10^{-4}$	$1\times 10^{-5}$
Mini-batch size	16	12
Maximum epochs	10	15
L2 regularization	$1\times 10^{-4}$	$1\times 10^{-4}$
Gradient threshold	5	5
Shuffle policy	Every epoch	Every epoch
Validation strategy	Validation during training; best validation loss retained	Validation during training; best validation loss retained
Early stopping patience	5 validation checks	5 validation checks
Data augmentation	Horizontal reflection; rotation ±10°; x/y translation ±10 pixels; scale 0.9–1.1	Horizontal reflection; rotation ±10°; scale 0.9–1.1
Training class balancing	Minority-class oversampling within training partition only	Minority-class oversampling within training partition only
Decision threshold for malignant class	0.5	0.5
Calibration method	Post hoc temperature scaling on source-domain validation set when eligibility criteria were met	Post hoc temperature scaling on source-domain validation set when eligibility criteria were met
Temperature bounds	0.5–5.0	0.5–5.0

CNN, convolutional neural network; ViT, vision transformer; ROI, region of interest. Input sizes follow architecture-specific pretrained baseline configurations and should not be interpreted as an isolated resolution comparison. Different mini-batch sizes and maximum epochs were used to accommodate the larger ViT input resolution and memory demand while maintaining stable fine-tuning. Hyperparameters were fixed before evaluation and were not optimized on external target datasets.

**Table 2:** Summary of selected public breast ultrasound datasets and their benchmark relevance.

Dataset	Reference	Images Used	Normal Images	Unique Patient IDs	ROI/Lesion Annotation	Benchmark Relevance
BUS-BRA	[9]	1875 lesion images	0	1875	Yes	Largest lesion-only dataset; near image-level patient granularity; useful as a high-sample training source
BrEaST	[10]	266 lesion images	4	256	Yes	Curated lesion-analysis dataset; useful small-source external-transfer condition
BUS-UCLM	[11]	264 lesion images	419	38	Yes	Structurally challenging dataset with aggregated patient identifiers; useful stress-test for patient-level splitting and dataset shift
BUSI	[12]	647 lesion images	133	780	Yes	Widely used public benchmark; includes sufficient normal images for auxiliary analysis

Normal images were excluded from the primary benign-versus-malignant classification task and were used only for auxiliary lesion-versus-normal confidence-based analysis when sufficient cases were available. ROI, region of interest.

**Table 3:** Overall internal and external performance and calibration summary by model and input mode.

Model	Input	Internal AUROC, Mean (95% CI)	External AUROC, Mean (95% CI)	Internal Balanced Accuracy, Mean (95% CI)	External Balanced Accuracy, Mean (95% CI)	Internal ECE Raw/Cal	External ECE Raw/Cal	Internal NLL Raw/Cal	External NLL Raw/Cal
CNN	ROI	0.859 (0.744–0.973)	0.735 (0.681–0.790)	0.776 (0.666–0.886)	0.648 (0.595–0.700)	0.135/0.136	0.133/0.124	0.500/0.494	0.637/0.611
CNN	Whole image	0.820 (0.619–1.000)	0.672 (0.621–0.723)	0.747 (0.560–0.934)	0.585 (0.536–0.633)	0.130/0.128	0.161/0.146	0.533/0.528	0.746/0.699
ViT	ROI	0.808 (0.646–0.970)	0.805 (0.762–0.848)	0.708 (0.547–0.870)	0.713 (0.671–0.755)	0.230/0.220	0.187/0.156	0.884/0.813	0.824/0.628
ViT	Whole image	0.719 (0.460–0.978)	0.666 (0.602–0.731)	0.663 (0.482–0.843)	0.594 (0.549–0.638)	0.254/0.247	0.241/0.175	1.124/1.060	1.184/0.789
Overall mean	—	0.801	0.719	0.723	0.635	0.187/0.183	0.180/0.150	0.760/0.724	0.848/0.682

The ‘Overall mean’ row reports the unweighted mean across the four model/input configurations in the table, with each configuration-level value based on scenario-level summaries averaged over five random seeds. AUROC, area under the receiver operating characteristic curve; CNN, convolutional neural network; ViT, vision transformer; ROI, region of interest; ECE, expected calibration error; NLL, negative log-likelihood; AUROC and balanced accuracy are reported as mean (95% CI); ECE and NLL are reported as raw/temperature-scaled; Cal, Calibrated; —, not applicable/not available.

**Table 4:** Best- and worst-performing external transfers ranked by mean external AUROC.

Rank	Train Dataset	Test Dataset	Model	Input	External AUROC	External Balanced Accuracy
1	BUS-BRA	BUSI	ViT	ROI	0.905	0.815
2	BUS-BRA	BUSI	CNN	ROI	0.896	0.815
3	BUS-BRA	BUS-UCLM	ViT	ROI	0.893	0.792
4	BUSI	BUS-UCLM	ViT	ROI	0.877	0.766
5	BUS-BRA	BUSI	CNN	Whole image	0.864	0.768
Lowest 1	BUS-UCLM	BUSI	ViT	Whole image	0.513	0.513
Lowest 2	BUS-UCLM	BUS-BRA	ViT	Whole image	0.538	0.514
Lowest 3	BUS-UCLM	BrEaST	CNN	Whole image	0.571	0.528
Lowest 4	BUS-UCLM	BUS-BRA	CNN	Whole image	0.574	0.524
Lowest 5	BrEaST	BUS-BRA	ViT	Whole image	0.586	0.549

AUROC, area under the receiver operating characteristic curve; CNN, convolutional neural network; ViT, vision transformer; ROI, region of interest.

**Table 5:** Sensitivity analysis excluding scenarios involving BUS-UCLM.

Model	Input	External AUROC, All Scenarios	External AUROC, Excluding BUS-UCLM	External Balanced Accuracy, All Scenarios	External Balanced Accuracy, Excluding BUS-UCLM
CNN	ROI	0.735	0.802	0.648	0.707
CNN	Whole image	0.672	0.718	0.585	0.630
ViT	ROI	0.805	0.824	0.713	0.737
ViT	Whole image	0.666	0.700	0.594	0.618

AUROC, area under the receiver operating characteristic curve; CNN, convolutional neural network; ViT, vision transformer; ROI, region of interest. Values are scenario-level aggregate means comparing all external scenarios with the subset excluding scenarios involving BUS-UCLM.

**Table 6:** Targeted ROI-margin sensitivity analysis under external dataset shift.

Model	ROI Margin	Mean External AUROC	Mean External Balanced Accuracy	AUROC *p* vs. 12%	Balanced Accuracy *p* vs. 12%
CNN	0%	0.778	0.655	0.375	1.000
CNN	10%	0.772	0.658	0.125	0.375
CNN	12%	0.751	0.656	Reference	Reference
CNN	20%	0.738	0.643	0.125	0.625
ViT	0%	0.834	0.706	0.625	0.875
ViT	10%	0.826	0.708	0.875	0.625
ViT	12%	0.816	0.698	Reference	Reference
ViT	20%	0.806	0.704	0.625	0.875

Values summarize four representative external transfer scenarios averaged over three random seeds. *p* values compare each alternative margin with the primary 12% margin using paired Wilcoxon signed-rank tests across matched source–target scenarios. AUROC, area under the receiver operating characteristic curve; CNN, convolutional neural network; ViT, vision transformer; ROI, region of interest.

## Data Availability

The source imaging datasets used in this study are publicly available from the original repositories cited in the manuscript. The reproducibility materials associated with the present benchmark comprise the implementation code, harmonized manifests, scenario-specific split files, seed-level outputs, aggregated summary tables, audit logs, and figure-generation scripts required to reproduce the reported analyses. These materials will be deposited in a public repository upon acceptance, and the repository link and versioned release identifier will be added to this statement before final publication. The released materials will permit exact reruns of the reported benchmark under the documented software environment, subject to continued availability of the source public datasets.

## Supplementary Materials

The following supporting information can be downloaded at: https://www.mdpi.com/article/10.3390/diagnostics16101537/s1, Table S1: Summary of dataset composition and lesion annotation availability, Table S2. Scenario-level paired comparison of ROI versus whole-image input under cross-dataset evaluation, Table S3. Calibration eligibility, audited safeguard checks, and context for the auxiliary lesion-versus-normal analysis, Table S4. Targeted ROI-margin sensitivity analysis under external dataset shift, Table S5. Paired comparison of alternative ROI margins against the primary 12% margin, Table S6. Contextual comparison with related breast ultrasound AI and medical-imaging AI studies.
