# Supplementary material for: Breast Ultrasound AI Under Dataset Shift: A Patient-Leakage-Aware Benchmark
Source: Diagnostics (Basel). 2026 May 19;16(10):1537. doi: 10.3390/diagnostics16101537 (PMC13206445; doi:10.3390/diagnostics16101537)
Supplement: Supplementary file 1 [file diagnostics-16-01537-s001.zip › diagnostics-4290933-supplementary.pdf]

Supplementary Materials

This appendix provides dataset-level context and benchmark audit information to support transparent interpretation of the main results. It summarizes dataset composition and patient-identifier structure, paired ROI-versus-whole-image comparisons under cross-dataset evaluation, and calibration eligibility, safeguard checks, and the scope of the auxiliary lesion-versus-normal confidence-based analysis. These materials clarify how dataset structure, calibration eligibility, and auxiliary-analysis availability influenced benchmark interpretation. It also reports targeted ROI-margin sensitivity results and paired comparisons of alternative ROI margins against the primary 12% margin. Table S6 provides a contextual comparison with related breast ultrasound AI, public-dataset, uncertainty-quantification, and medical-imaging AI translation studies to clarify how the present benchmark differs from prior performance-focused studies.

Table S1. Summary of dataset composition and lesion annotation availability.

Dataset	Total images/rows	Lesion images used for main benign–malignant task	Normal images reserved for auxiliary analysis	Unique patient IDs	ROI / lesion annotation available
BUS-BRA	1875	1875	0	1875	Yes
BUS-UCLM	683	264	419	38	Yes
BUSI	780	647	133	780	Yes
BrEaST	270	266	4	256	Yes

ROI, region of interest.

Table S2. Scenario-level paired comparison of ROI versus whole-image input under cross-dataset evaluation.

Model family	Comparison	Mean paired difference (ROI – whole image)	95% CI	Statistical test	p-value	Interpretation
CNN	External AUROC	0.064	0.025 – 0.102	Wilcoxon signed-rank	0.009	ROI input improved external discrimination for the CNN baseline
CNN	External balanced accuracy	0.063	0.039 – 0.087	Wilcoxon signed-rank	<0.001	ROI input improved threshold-based external performance for the CNN baseline
ViT	External AUROC	0.139	0.101 – 0.176	Wilcoxon signed-rank	<0.001	ROI input produced the largest external discrimination gain for the ViT baseline
ViT	External balanced accuracy	0.119	0.100 – 0.138	Wilcoxon signed-rank	<0.001	ROI input improved threshold-based external performance for the ViT baseline

CNN, convolutional neural network; ViT, vision transformer; ROI, region of interest.

Table S3. Calibration eligibility, audited safeguard checks, and context for the auxiliary lesion-versus-normal analysis.

Category	Item	Value	Benchmark relevance
Overall benchmark execution	Completed train-test runs	320	Total number of completed benchmark runs across internal and cross-dataset settings, random seeds, input modes, and model families
Temperature-scaling eligibility	Internal runs meeting predefined validation-set eligibility criteria	44 / 80 (55.0%)	Shows that post hoc calibration could not be applied uniformly across all internal runs
Temperature-scaling eligibility	External runs meeting predefined validation-set eligibility criteria	180 / 240 (75.0%)	Shows broader calibration eligibility under cross-dataset evaluation than under internal testing
BrEaST-source ROI	Seeds meeting calibration eligibility	2 / 5	Indicates limited calibration eligibility in a small-source setting
BrEaST-source ROI	Seeds with the temperature parameter fixed at 1.0	3 / 5	Indicates that temperature scaling was not estimable in all seeds under the predefined safeguards
BrEaST→BUSI ROI	Lesion-containing BUSI test cases	647	Size of the lesion-containing external test set used in the audited scenario
BrEaST→BUSI ROI	Auxiliary normal BUSI cases	133	Size of the auxiliary normal-image set used for uncertainty analysis
BrEaST→BUSI ROI	Patient overlap between lesion-containing and auxiliary normal partitions	None	Supports cleaner interpretation of the auxiliary lesion-versus-normal uncertainty analysis
BrEaST→BUS-UCLM ROI	Lesion-containing BUS-UCLM test cases	264	Size of the lesion-containing external test set used in the audited scenario
BrEaST→BUS-UCLM ROI	Auxiliary normal BUS-UCLM cases	419	Size of the auxiliary normal-image set used for uncertainty analysis
BrEaST→BUS-UCLM ROI	Relation between lesion-containing and auxiliary normal partitions	Not patient-disjoint	Requires caution in interpreting the lesion-versus-normal uncertainty results
Auxiliary uncertainty-analysis scope	Datasets with sufficient normal cases for analysis	BUSI and BUS-UCLM	Explains why auxiliary lesion-versus-normal results were narrower in scope than the primary benchmark
Auxiliary uncertainty-analysis interpretation	Intended role of lesion-versus-normal uncertainty analysis	Auxiliary safety-oriented assessment, not a definitive open-set benchmark	Clarifies the intended interpretation of the uncertainty-based analysis

ROI, region of interest.

Table S4. Targeted ROI-margin sensitivity analysis under external dataset shift.

Model	ROI margin	AUROC	Balanced accuracy	ECE	NLL
CNN	0%	0.778	0.655	0.112	0.580
CNN	10%	0.772	0.658	0.109	0.590
CNN	12%	0.751	0.656	0.121	0.595
CNN	20%	0.738	0.643	0.137	0.664
ViT	0%	0.834	0.706	0.154	0.783
ViT	10%	0.826	0.708	0.155	0.846
ViT	12%	0.816	0.698	0.173	0.880
ViT	20%	0.806	0.704	0.143	0.733

Values are means across four representative external source-target scenarios after averaging across three random seeds. AUROC, area under the receiver operating characteristic curve; ECE, expected calibration error; NLL, negative log-likelihood; CNN, convolutional neural network; ViT, vision transformer; ROI, region of interest.

Table S5. Paired comparison of alternative ROI margins against the primary 12% margin.

Model	Comparison	Mean AUROC difference	Mean balanced accuracy difference	AUROC p	Balanced accuracy p
CNN	0% - 12%	+0.0268	-0.0004	0.375	1.000
CNN	10% - 12%	+0.0207	+0.0026	0.125	0.375
CNN	20% - 12%	-0.0131	-0.0122	0.125	0.625
ViT	0% - 12%	+0.0177	+0.0084	0.625	0.875
ViT	10% - 12%	+0.0097	+0.0102	0.875	0.625
ViT	20% - 12%	-0.0095	+0.0059	0.625	0.875

Differences are calculated as alternative margin minus 12% margin across matched source-target scenarios. Wilcoxon signed-rank p values are reported; no comparison reached statistical significance. AUROC, area under the receiver operating characteristic curve; CNN, convolutional neural network; ViT, vision transformer.

Table S6. Contextual comparison with related breast ultrasound AI and medical-imaging AI studies.

Reference	Dataset(s) or evidence base	Main method or focus	Task / evaluation emphasis	Reported performance context	Relevance to the present benchmark
[1]	Systematic review of AI-enhanced handheld breast ultrasound screening studies	Review of diagnostic-test accuracy studies	Breast ultrasound screening and diagnostic accuracy	Reported evidence supports the promise of AI-assisted breast ultrasound, but study designs and validation settings vary across included reports	Supports the clinical relevance of breast ultrasound AI, while reinforcing the need for externally validated and reproducible evaluation
[2]	Review of AI applications in breast imaging	Clinical review of AI in breast imaging	Diagnostic workflow, clinical application, and translational challenges	Highlights increasing use of AI in breast imaging but also emphasizes challenges for clinical adoption	Provides clinical context for why robustness, calibration, and workflow relevance are important
[3]	Review of breast cancer AI methods	Review of augmentation, segmentation, diagnosis, and prognosis methods	Broader breast cancer imaging AI	Summarizes strong reported model performance across breast-imaging tasks, but across heterogeneous datasets and study designs	Supports the need to distinguish model-development performance from reproducible external benchmark performance
[4]	Systematic review of breast ultrasound AI research	Review of breast ultrasound AI advances	Breast ultrasound classification, segmentation, diagnosis, and decision support	Reports rapid progress in breast ultrasound AI, with many studies emphasizing model performance within selected datasets	Provides direct literature context for the present breast ultrasound benchmark and motivates more rigorous leakage-aware evaluation
[5]	Breast ultrasound lesion-classification datasets	Transfer learning, fine-tuning, and inter-/extra-lesion tissue analysis	Lesion classification and role of peri-lesional context	Reported that tissue context around lesions can influence breast-lesion classification robustness	Supports the rationale for comparing whole-image and ROI-based input and for testing ROI-margin sensitivity
[6]	Breast ultrasound diagnostic data with multiple tumoral regions	Multi-task transformer with local-global feature interaction and multiple tumoral-region guidance	Transformer-based breast cancer diagnosis	Reported strong transformer-based diagnostic performance using region-guided modeling	Supports inclusion of a ViT baseline and supports discussion of architecture–input-representation interaction
[7]	Multi-society radiology AI statement	Practical guidance for AI development, implementation, and monitoring	Clinical translation, governance, monitoring, and deployment	Emphasizes that AI tools require careful validation, monitoring, and governance before clinical deployment	Supports the manuscript's interpretation that benchmark performance alone is not proof of deployment readiness
[8]	Review of medical-imaging AI applications	Assessment of internal validity and external validation limitations	External validation and scope of medical-imaging AI studies	Shows that many medical-imaging AI applications demonstrate high internal validity but limited external validation	Directly supports the present study's emphasis on cross-dataset testing rather than single-dataset performance

Reference	Dataset(s) or evidence base	Main method or focus	Task / evaluation emphasis	Reported performance context	Relevance to the present benchmark
[9]	BUS-BRA	Public breast ultrasound dataset for computer-aided diagnosis	Dataset resource for breast ultrasound CAD research	Provides a large public lesion-image dataset with near-image-level patient granularity	Included as a major source/target dataset and high-sample training source in the present benchmark
[10]	BrEaST	Curated benchmark dataset for ultrasound-based breast lesion analysis	Dataset resource for lesion analysis	Provides curated lesion-analysis data with ROI/lesion information	Included as a smaller curated dataset to test external-transfer behavior under limited source-size conditions
[11]	BUS-UCLM	Breast ultrasound lesion segmentation dataset	Dataset resource with lesion annotations and normal images	Provides a structurally distinctive dataset with aggregated patient identifiers and substantial normal-image availability	Used as a challenging stress-test dataset; results involving BUS-UCLM were interpreted cautiously because of its patient-identifier structure
[12]	BUSI	Public breast ultrasound image dataset	Benign, malignant, and normal breast ultrasound images	Widely used public benchmark dataset for breast ultrasound AI studies	Included as a major target/source dataset and as one of the datasets with sufficient normal cases for auxiliary analysis
[13]	Review/resource paper on public ultrasound datasets and models	Public dissemination and open sourcing of ultrasound datasets and deep-learning resources	Dataset availability and reproducibility	Highlights increasing availability of public ultrasound data and models	Supports the use of public datasets for reproducible benchmarking while acknowledging public-data limitations
[14]	Review of uncertainty quantification in medical-image analysis	Trustworthy clinical AI and uncertainty quantification	Calibration, uncertainty, and reliability	Emphasizes that uncertainty and reliability metrics are important for trustworthy clinical AI	Supports the inclusion of calibration, expected calibration error, negative log-likelihood, and confidence-based auxiliary analysis
[15]	Review of uncertainty quantification in medical-image analysis	Probabilistic and non-probabilistic uncertainty methods	Uncertainty estimation and reliability	Highlights the limitations of discrimination-only evaluation in medical imaging AI	Supports the manuscript's interpretation that calibration complements, but does not replace, external validation
[16]	Review/guidance on AI and machine learning in medical imaging	Development-to-translation considerations	Model development, validation, and clinical translation	Emphasizes that development performance must be followed by validation and translational assessment	Supports the manuscript's cautious framing that the benchmark is not evidence of clinical deployment readiness

This table is intended as a contextual comparison rather than a direct numerical meta-analysis. Direct performance comparison across studies is limited because prior reports differ in dataset selection, preprocessing, task definition, train-test splitting strategy, patient-level leakage control, model architecture, threshold selection, and use of internal versus external validation. CAD, computer-aided diagnosis; ROI, region of interest; AI, artificial intelligence.
